# Emulsion Gels as Precursors for Porous Silicones and All-Polymer Composites—A Proof of Concept Based on Siloxane Stabilizers

**DOI:** 10.3390/gels8060377

**Published:** 2022-06-14

**Authors:** Carmen Racles, Adrian Bele, Ana-Lavinia Vasiliu, Liviu Sacarescu

**Affiliations:** 1 Department of Inorganic Polymers, “Petru Poni” Institute of Macromolecular Chemistry, Aleea Gr. Ghica Voda 41A, 700487 Iasi, Romania; bele.adrian@icmpp.ro (A.B.); livius@icmpp.ro (L.S.); 2 Mihai Dima Laboratory of Functional Polymers, “Petru Poni” Institute of Macromolecular Chemistry, Aleea Gr. Ghica Voda 41A, 700487 Iasi, Romania; vasiliu.lavinia@icmpp.ro

**Keywords:** amphiphilic siloxanes, emulsion gels, polysiloxanes, porous silicones, xerogels, negative interfacial tension, emulsion templating

## Abstract

In spite of its versatility, the emulsion templating method is rather uncommon for the preparation of porous silicones. In this contribution, two siloxane-containing stabilizers, designed to be soluble in polar (water) and non-polar (toluene) solvents, respectively, were used in low concentrations to produce stable emulsions, wherein polysiloxane gels were obtained by UV-photoinitiated thiol-ene click cross-linking. The stabilizers exhibited negative interfacial tension, as measured by Wilhelmy plate tensiometry. The emulsion gels evolved into porous silicones (xerogels), with tunable morphology and properties. According to TEM and SEM investigations, the emulsion template was preserved in the final materials. Several parameters (e.g., the structure of the polysiloxane precursors, composition of the emulsion gels, nature of the continuous phase, cross-linking conditions, or additives) can be varied in order to obtain porous elastic materials with desired properties, such as Janus membranes, absorbent monoliths, all-polymer porous composites, or silicone-swollen gels. The feasibility of these types of materials was tested, and exemplary porous silicones were briefly characterized by contact angle measurements, mechanical testing, and absorption tests. The proposed method is simple, fast, and economic, uses very little amounts of stabilizers, and can be adjusted as a green technique. In this contribution, all the silicon-based materials with a convenient design were prepared in house.

## 1. Introduction

Emulsion templating is one of the most important methods for producing porous materials. It generally involves reactions that take place in the external (continuous) phase, and lead to monoliths. A lot of techniques and experimental setups, as well as various chemical reactions, have been demonstrated for production of a wide range of porous materials, including polymers, organic–inorganic hybrids or composites, inorganic structures, or porous carbon materials [1]. Several types of emulsions may form by proper choice of the system components and concentration, and these can be polymerized to form porous materials with tunable morphology. For example, complex emulsion structures have been obtained [2] by using osmotically driven water–oil phase separation. 

In particular, emulsion gels are two-phase systems in which the continuous phase is a gel, and serve as templates for highly porous materials. In most cases, the disperse phase consists in oil droplets dispersed in a hydrogel (or a biopolymer gel), and these are widely used in food products [3]. The reverse combinations of organic gels with high water content as the internal phase are precursors for low density, porous materials [4]. As in the general case of emulsions, the preparation of emulsion gels involves the use of a stabilizer, which is basically a surfactant, having the role of reducing the interfacial tension between the two phases (dispersed phase and dispersion medium) to increase the kinetic stability [5].

Porous polymers based on cage-like organosiloxanes as building blocks are highly ordered materials with interesting applications, such as adsorption, sensing, catalysis, and drug delivery [6]. On the other hand, porous silicones derived from linear polysiloxanes are relatively scarcely described in the literature, possibly due to expected alterations of their mechanical characteristics as compared with non-porous similar materials. However, they can also be valuable materials for special applications. For example, polydimethylsiloxanes (PDMS) with 3D interconnected pores have been prepared using sugar cubes or salt crystals as templates [7,8]. The resulted lightweight materials showed interesting flexoelectric properties, being suitable for applications as wearable sensors. By infiltration of multilayer graphene nanoplatelets, electrically conductive porous PDMS have been prepared [9]. 

The emulsion templating procedure has been occasionally used to produce porous silicones, for example, as acoustic metamaterials [10]. In this case, the continuous phase consisted of a UV-curable epoxy silicone mixture, and low to medium water internal phase fractions were introduced. For the same purpose, a hydrosilylation reaction was used in the emulsion templating process [11], and low sound speed was reported even for low porosity. Commercially available silicone precursors and surfactants (of the polyethylene oxide–polysiloxane type) were used in both cases. Direct emulsion of silicone in water was also used to produce porous silicones, wherein the cross-linking was done by polyaddition or polycondensation [12]. In other situations, PDMS was added to pre-formed porous polymer materials or gel compositions, in order to improve their properties, for example, the flexibility, when used as a cross-linker [5], or self-cleaning and self-repairing abilities, when used as a lubricating liquid [13]. 

While ultra-low surface tension is a well-documented phenomenon with practical use in oil recovery [14], the concept of negative interfacial tension was under debate over 100 years ago [15,16]. Mathematical models and simulations, as well as physics approaches, indicate that negative surface and interfacial tension are possible [17,18], but only for nonequilibrium macroscopic solutions or for equilibrium nano-solutions [19]. It was inferred that “in microemulsion formation the initial negative interfacial tension is probably the main driving force, even in nonionic systems” [18]. However, rather few studies actually demonstrate this phenomenon experimentally. One such case refers to interfacial tension between miscible solvents [20]. The concept of monodispersed microdroplets formed at the interface and their shape transformation induced by negative interfacial tension was accepted [21] as an explanation for the formation of “smectic LC vesicle structures analogous to the biomembranes in living systems”. It was stated that, most probably, the negative interfacial tension is due to the formation of a monolayer of surfactant at the interface [22].

Thus, as mentioned, surfactants are key elements in emulsion preparation. A very particular case is that of siloxane surfactants. These contain dimethylsiloxane units as the hydrophobic part and have several characteristics that make them unique and valuable compounds [23]. For example, they are able to considerably reduce the surface tension of water and organic media, present very low critical micelle concentration, have high ability of spreading and wetting, and tend to form vesicles in aqueous solution [23,24]. Besides the most commonly employed class, which are the polyether–siloxane surfactants of various design, a significant number of newer and less investigated surface-active silicones has been reported, wherein the hydrophilic groups can be nonionic (e.g., carbohydrates), ionic, or zwitterionic, and attached in terminal, pendant, or gemini architectures [25,26]. 

The solubility and the solution properties of siloxane surfactants within a certain class depend exclusively on the length of the siloxane segment and can be tuned by synthesis. For a given hydrophilic functionality, the hydrophilic–lipophilic balance (HLB) may be designed for applications spanning from water-in-oil to oil-in-water emulsions, while their surface activity may manifest in aqueous or organic solutions. 

We have reported several new types of siloxane-based amphiphilic compounds, and among them, pyridyl-modified siloxanes showed remarkable potential. They proved to be surface-active in water or in organic solvents, depending on the length of the dimethylsiloxane segment, and could manifest phase transfer ability, besides other properties [27]. 

Herein, we explore the potential of two pyridyl-modified siloxane stabilizers, specially designed to be soluble in water or toluene, for emulsion templating of silicone gels, aiming to produce porous monoliths or films, with a minimum amount of additives. These surfactants showed negative interfacial tension and thus can promote quasi-spontaneous emulsification. The photo-induced cross-linking of vinyl-polysiloxanes offers a fast, clean, and simple alternative for the preparation of stable, porous silicone materials, and is also a tool for better understanding the complexity of the water/toluene emulsion system containing the pair of in-house prepared surfactants. The main purpose of this research was to test the feasibility of the emulsion templating method, adapted for dilute solutions of these surfactants and using thiol-ene click photo-addition cross-linking, in preparation of porous silicones. Several variations, aiming to tune the characteristics of the final materials, were identified. For example, the transient gel phase can be preserved for a long time, especially when the toluene solvent is replaced by less volatile octamethylcyclotetrasiloxane (D4). The addition of water-soluble polymers allows one-step preparation of composite porous materials. Moreover, many parameters can be tuned to obtain optimized materials, according to envisaged applications.

## 2. Experimental

### 2.1. Materials 

Trimethylolpropane tris(3-mercaptopropionate) (SHCL), octamethylcyclotetrasiloxane (D4), poly(ethylene glycol) 12,000 g/mol (PEG), as well as all the reagents necessary for the synthesis of precursors and stabilizers were purchased from Sigma-Aldrich, St. Louis, MO, USA. The photo-initiator 2,2-dimethoxy-2-phenylacetophenone (DMPA) was supplied by Merck. The solvents were purchased from Chemical Company (Iasi, Romania), and double-distilled water was used in all experiments.

The stabilizers (D and T) were synthesized as previously described [27] by the addition of 4-aminopyridine to epoxy-terminated siloxane oligomers. 

Dimethylmethylvinylsiloxane copolymers with 8.5 (Co-8.5) and 1.8 (Co-1.8) mol% vinyl-substituted units, having similar molecular weights (M_n_ of around 50,000 g/mol) were prepared by cation exchanger catalyzed ring opening copolymerization of octamethylcyclotetrasiloxane (D4) and tetramethyl-tetravinylcyclotetrasiloxane (V4) [28]. A long oligomer with vinyl chain ends and M_n_ ca. 10,000 g/mol (O) was obtained by the same procedure, using D4 as monomer and 1,3-bis(divinyl)tetramethyldisiloxane as chain transfer agent. 

### 2.2. Methods

Surface, interfacial, and continuous surface tension measurements were done with a Sigma 700 automated tensiometer from KSV, using the Wilhelmy plate method. For interfacial tension measurements, special care was taken for accurate detection of the interface, including staining of the water layer with methylene blue.

The TEM analysis of the samples was performed using the Hitachi HT-7700 equipment operated at 100 kV accelerating voltage. This instrument is dedicated to the study of soft materials and allows observation of details in high magnification and good contrast conditions. Samples of the studied emulsions were drop cast onto TEM grids (300 mesh, copper grids, Ted Pella). Excess of the liquid was blotted away and the samples were let to dry overnight in a vacuum oven at 70 °C. Inspection of the samples was conducted after cooling the samples chamber at −170 °C. 

For SEM investigations, depending on the sample, two microscopes were used: Quanta 200 (FEI Company, Brno, Czech Republic), using a LFD (Large Field Detector), working in low vacuum mode at 20 kV without any coating, and Verios G4 UC (Thermo Scientific, Waltham, MA, USA), using a CBS (Concentric Backscatter Detector. Brno, Czech Republic) at 5 kV, in which case the samples were coated with 6 nm of platinum with a Leica EM ACE200 Sputter coater to provide electrical conductivity and to prevent charge buildup during exposure to the electron beam. All the materials were analyzed in cross-section obtained by breaking or cutting with scissors.

Fourier transform infrared (FT-IR) spectra were registered on a Bruker Vertex 70 Spectrometer in transmittance mode, in the 400–4000 cm^−1^ spectral range, with a resolution of 4 cm^−1^ and accumulation of 32 scans.

The contact angle was measured with a DSA25 Drop Shape Analyzer (Kruss), in the Sessile Drop mode, using water droplets of 1 µL. The values of the contact angle are the mean of a minimum of 3 determinations.

Mechanical measurements were made using an Instron 3365, two-column universal mechanical testing machine. The stress–strain measurements were made at an extension rate of 50 mm/min, at room temperature (laboratory conditions). All samples were measured two times and the average of the obtained values was reported. The compression tests were done for monolith samples at 10 mm/min compression rate and different compressive strains (10%, 30%, and 50%). 

Swelling experiments were done by immersing pieces of porous silicone materials in the solvents for 30 min and draining and weighing the swollen samples. The absorption capacity (g/g) was calculated with the formula: (m − m_0_)/m_0_, where m_0_ and m represent the weight of the sample before and after absorption of the solvent. 

### 2.3. Preparation of Emulsion Gels and Xerogels

In preliminary experiments (PE1-3), in a dilute toluene solution of T, a dimethylmethylvinyl siloxane copolymer with 8.5% vinyl groups (copolymer concentration 20% *w*/*v*), the cross-linker (15% *w*/*w* reported to the copolymer) and photoinitiator DMPA (2.4%) were dissolved. Distilled water was added (10% or 20% reported to toluene) with or without PEG dissolved, and the mixture was processed with a SilentCrusher Homogenizer (Heidolph) at 14,000 rpm. Then, the mixture was either poured in a Teflon vessel or kept in an open vial, and irradiated with a laboratory lamp (Herolab) at 365 nm for 10–15 min. The materials were allowed to dry freely in an ambient atmosphere.

In the optimized experiments (EG1-10), 1% toluene solution of T (typically 2.4 mL) was mixed with 1% water solution of D (according to Table 1), by manual shaking and sonication for 30 s in an ultrasonic cleaning bath (Elmasonic P, Elma, Singen, Germany, at 37 kHz and 90% power). Then, the silicone precursor (0.5 g) and the cross-linker SHCL (in equimolar ratio reported to vinyl groups) were added to the formed emulsion and dissolved by manual shaking and sonication for 30 s in the cleaning bath. The photoinitiator (DMPA, 12 mg) was added, and after gentle agitation, the mixture was submitted to UV irradiation with a 400 W Phillips UV-A Lamp, for 3–5 min. The materials were rinsed with water several times to remove excess SHCL and other impurities, and allowed to dry freely in ambient atmosphere, or, alternatively, were supplementary dried in a vacuum oven at 70 °C for 24 h. 

The composition of all the samples is described in Table 1. In all cases, the ratio between the vinyl–silicone, the cross-linker, and the catalyst was kept constant. For sample EG9, the stabilizer T was first dissolved in D4 (instead of toluene), with the aid of a drop of chloroform and then the entire protocol described above was followed.

## 3. Results and Discussion 

### 3.1. Surface Properties of the Stabilizers

**Scheme 1:**
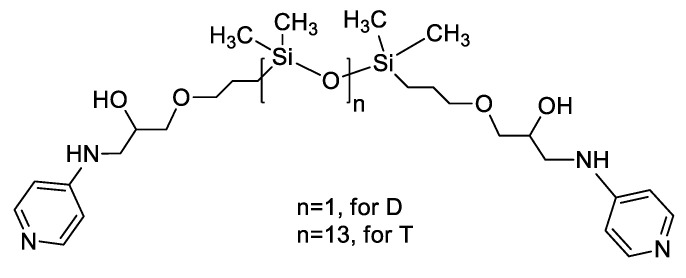
Structure of the siloxane stabilizers.

Two siloxane compounds bearing pyridyl terminal groups and having one (disiloxane, D) or 13 (telechelic, T) siloxane units were prepared according to our previous report [27] (Scheme 1). These compounds, with similar structure, are soluble in water and nonpolar organic solvents (toluene), respectively. Surface activity in aqueous solution was demonstrated for D. A very close homologue of T showed remarkable phase transfer ability of organic molecules from water into chloroform by the formation of water-in-chloroform (micro)emulsions [27]. 

In this contribution, we further explored the surface properties of these modified siloxanes (Table 2). They have very different HLB values [27] that are 12 for D and 5 for T, which indicate applications in direct (o/w) or reverse (w/o) emulsions, respectively. First, their interfacial tension against complementary liquids was measured by the Wilhelmy plate method. By this method, we found small negative values, i.e.,: −4.2 mN/m for an aqueous solution of D versus toluene, and −2.95 mN/m for a toluene solution of T versus water. The plausible explanation of the observed phenomenon is the strong amphiphilic character of the siloxane surfactants, which promotes spontaneous distribution of these molecules within the interfacial region. Repeated measurements with care for accurate detection of the interface, including staining of the water layer with methylene blue were done. In addition, interfacial tension was also measured for D with a continuous surface tension module, providing the appropriate parameters setup. It was observed that the values obtained for different concentrations of the D solutions were close to those measured in the usual surface/interfacial tension mode (Figure S1). 

**Figure 1:**
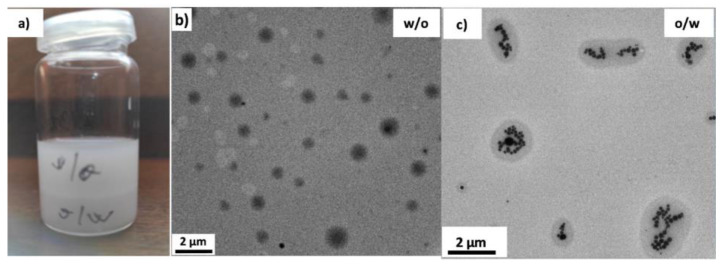
Image (**a**) and morphology of the emulsion obtained with 1 g/L toluene solution of T and water; (**b**) TEM image of the w/o emulsion; (**c**) TEM image of the o/w emulsion.

The surface tension of the emulsions formed with each surfactant was also measured (Table 2). The mean values for the toluene-in-water (o/w) emulsions in both cases, as well as for the water-in-toluene (w/o) phase in the case of T surfactant (Figure 1), were very close, around 26 mN/m. However, the standard deviation was rather high for the emulsion formed by D, which indicates poorer stability. 

### 3.2. Morphology of the Emulsions and Templated Xerogels

Negative interfacial tension values are correlated with the spontaneous formation of microemulsions. In our case, formation of an emulsion at gentle manual shaking was indeed observed. Depending on the water content and on the nature and level of the external energy, the emulsification process occurred differently. First we focused our attention on the organic-soluble T surfactant in contact with water. Under weak agitation, two types of emulsions formed (Figure 1), which is in line with the previous observation of phase transfer ability. 

The TEM investigations revealed completely different morphology in the two emulsions (Figure 1b,c). The w/o macroemulsion has large dimensions of the internal phase domains, within 400 nm–1 mm range, while the o/w emulsion presents particles of 100–200 nm assembled in a composite morphology. Interestingly, a completely different situation occurred when a high-energy ultrasonication was applied, using an ultrasonic processor (VC 505) (Figure S2). This time both emulsions formed, but the o/w emulsion was much more stable. Moreover, its milky aspect indicates a macroemulsion.

**Scheme 2:**
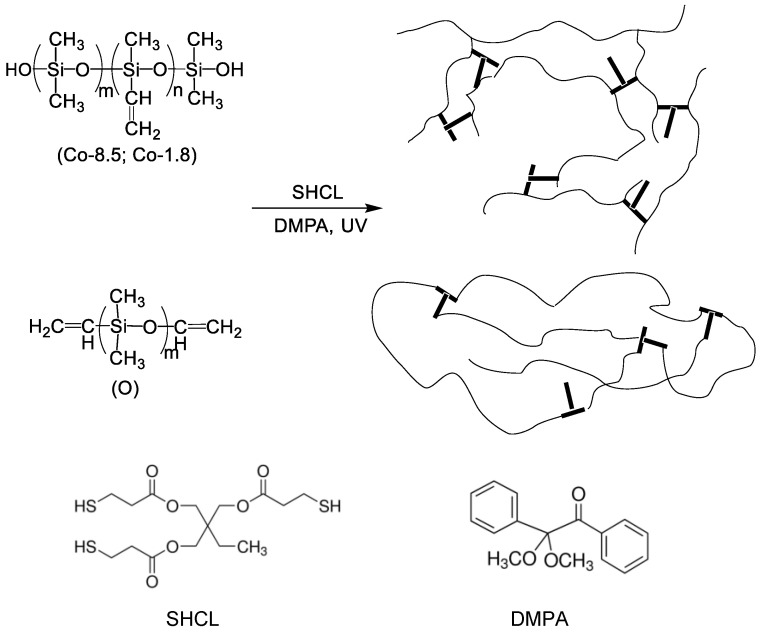
Photo-initiated thiol-ene cross-linking reactions of vinyl silicones, and structures of the cross-linker (SHCL) and the photoinitiator (DMPA).

To prepare the porous silicones, a UV-photoinitiated cross-linking reaction was used, which is a thiol-ene addition between a vinyl-containing polysiloxane and a trifunctional thiol (SHCL), as depicted in Scheme 2. This reaction is well known in organic chemistry [29,30], and also employed in silicones chemistry [23,31,32,33], as a quick (click), clean process, allowing functionalization or cross-linking of polysiloxanes. 

**Figure 2:**
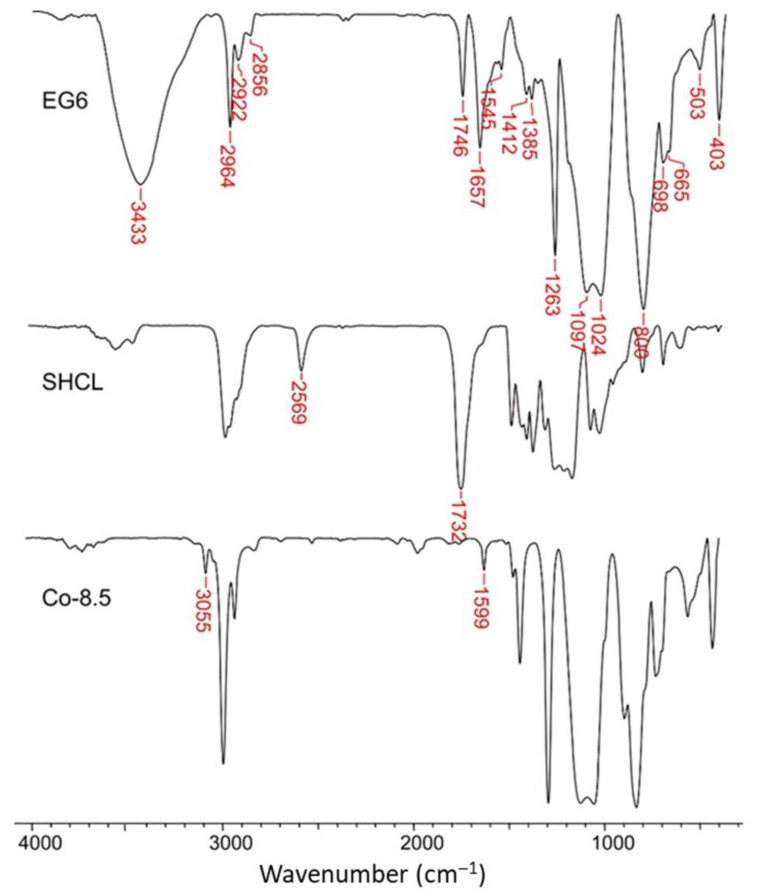
FT-IR spectra of Co-8.5, SHCL, and the reaction product (EG6).

The completion of the cross-linking reaction can be observed in the FT-IR spectrum of material EG6, compared with the starting vinyl–silicone Co-8.5 and the SHCL (Figure 2). In the spectrum of the initial dimethylmethylvinylpolysiloxane (Co-8.5), the absorption bands corresponding to vinyl groups are located at 1600 and 3055 cm^−1^, while the bands at 1263 cm^−1^, 1097 cm^−1^, 1024 cm^−1^, and 800 cm^−1^ are characteristic of polysiloxanes. The thiol absorption band in SHCL appears at 2569 cm^−1^, and the band at 1732 cm^−1^ is assigned to the ester groups. In the cross-linked material, neither the vinyl nor the thiol bands are present anymore, and additional bands coming from the surfactants are visible at 1657 cm^−1^ and 3433 cm^−1^ (secondary amine and adsorbed water) [27]. The polysiloxane signature remained unchanged, while the ester band suffered a slight shift due to changes in H-bonding. 

**Figure 3:**
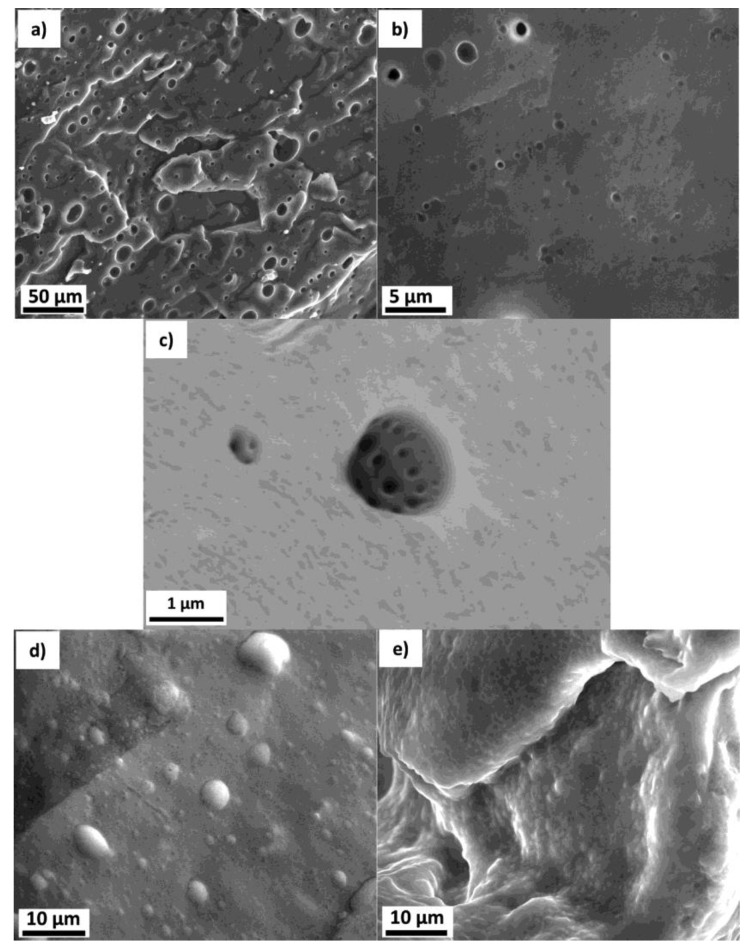
SEM images showing the morphology of porous silicones obtained without co-surfactant, from a dilute solution of T, emulsified with 10% water ((**a**,**b**)—sample PE1), 20% water ((**c**)—sample PE2-observed through atomic number contrast detector); and 10% water containing PEG ((**d**,**e**)—sample PE3).

When the dilute toluene solution of T was used as a continuous phase, having a vinyl-containing siloxane copolymer dissolved, together with an SH-containing cross-linker and the photoinitiator, a low amount of water was introduced as the internal phase (10–20%). The mixture was submitted to UV irradiation and the obtained gels (Figure S3) were dried in ambient conditions before SEM analysis. As can be observed in Figure 3, porous materials were obtained, presenting a “double emulsion structure” [2] with both large (micron size) pores distributed on a “sea island” pattern and small (submicron) ones, as well as combinations of these (Figure 3c). A similar composition, wherein PEG was dissolved in the water phase, gave a polymer–polymer composite. Its SEM images (Figure 3d,e) clearly show micron size PEG particles (instead of the large pores in the previous example) and small pores (Figure 3d). The observed morphology of this composite material confirms the fact that the initial emulsion served as a template, since PEG particles are indeed formed in the w/o domains. 

**Figure 4:**
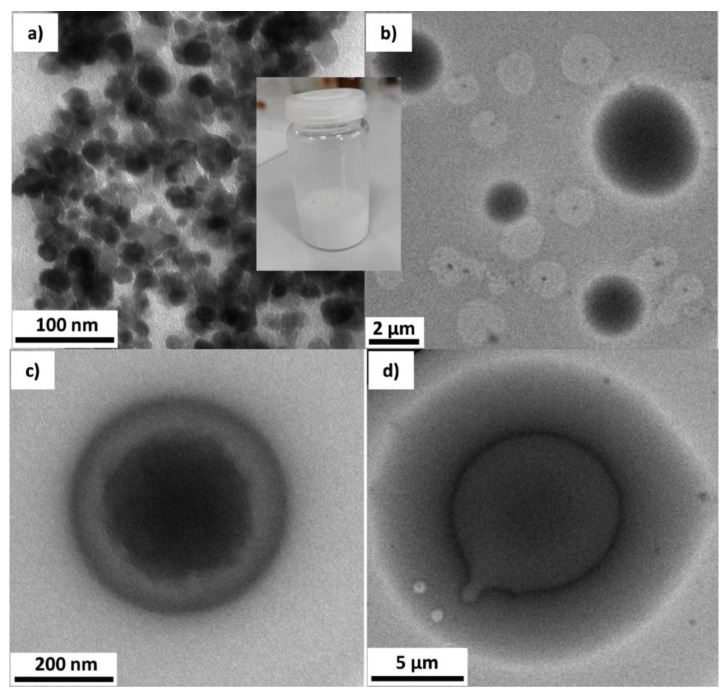
The macroscopic aspect and TEM images of an emulsion of 1% T (in toluene) and 1% D (in water), f = 0.5, after manual shacking and sonication in a cleaning bath. Several types of structures were identified in the same sample: o/w (**a**), w/o (**b**), o/w/o (**c**), and w/o/w (**d**).

In the next step, the formulation was changed, by addition of D (1%) in the aqueous phase as co-surfactant. This allowed us to obtain stable emulsions just by gentle manual mixing, while short time sonication in an ultrasound bath additionally increased the stability and homogeneous aspect. The complex composition of the as-obtained emulsion can be visualized in Figure 4. 

Several types of disperse objects were observed by TEM, corresponding to double emulsions (w/o and o/w), as well as to triple emulsions (o/w/o and w/o/w), and overall a large polydispersity of size and shape. Nevertheless, the emulsion was stable enough, as to preserve the morphology much longer than the timeframe of the UV cross-linking of the polysiloxane mixture. Since both toluene-based and water-based emulsion patterns were detected, a logical assumption is that a bicontinuous phase forms during sonication, allowing coexistence of all these aggregates. 

**Figure 5:**
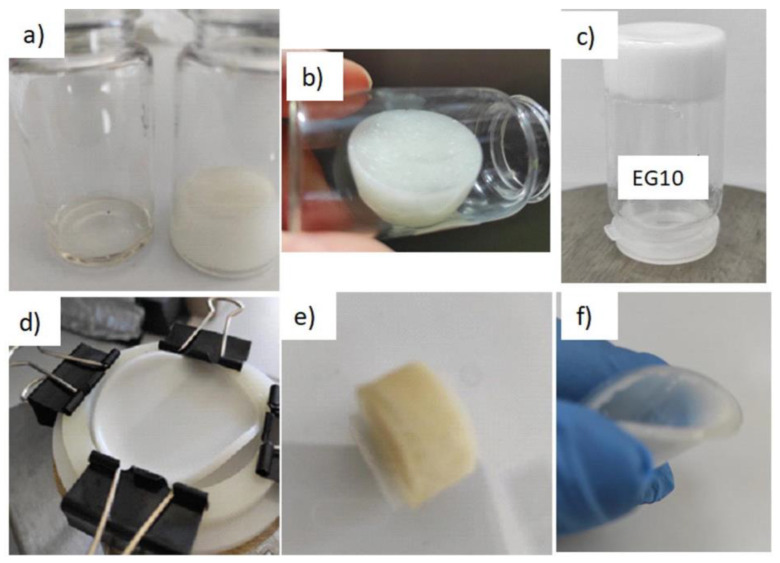
Silicone emulsion gels with 33% or 50% water phase: (**a**) comparison between the amount of silicone used and the corresponding gel; (**b**,**c**) swollen gels; (**d**) swollen film just after UV irradiation; (**e**) dried monolith; (**f**) flexible dried film.

With the surfactants pair, silicone gels with 33–50% water content could be easily obtained (Figure 5), which were processed as monoliths or thick films after drying in ambient conditions. 

**Figure 6:**
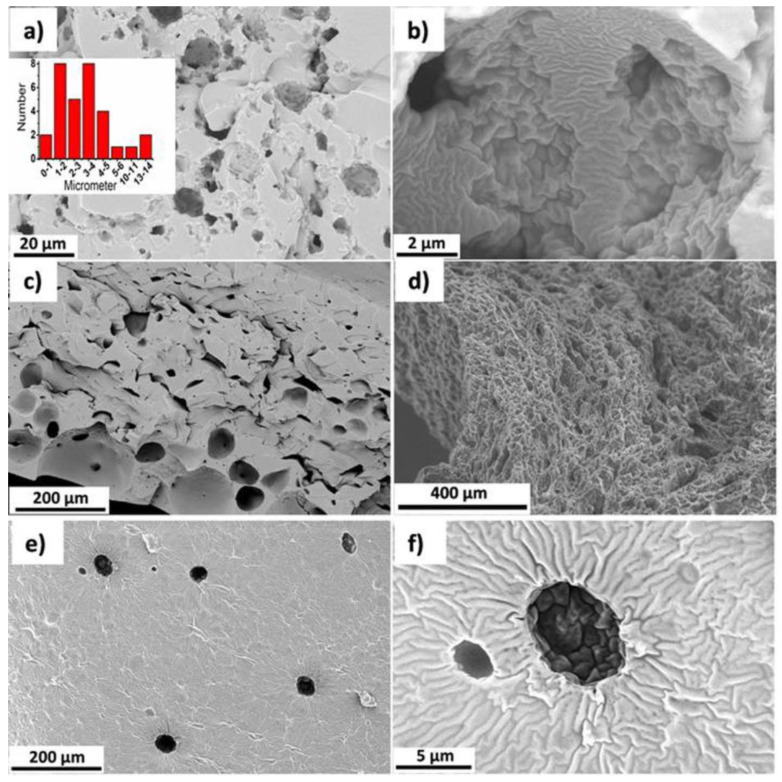
SEM images showing complex morphology of the xerogels in the case of medium internal phase content: multiple emulsion structure and histogram of pores size for f = 0.25 ((**a**,**b**)—sample EG2), gradient emulsion structure for f = 0.33 ((**c**)—sample EG5), highly porous monolith for f = 0.5 ((**d**)—sample EG10M), and phase separated film from the emulsion with f = 0.8 ((**e**,**f**)—sample EG7).

For f = 0.25, the multiple emulsion structure [2] was observed by SEM in the xerogel (Figure 6a,b). Additional elements were a thin smooth layer on the surface, which is due to evaporation of toluene during heat-generating UV irradiation, and the wrinkled walls which result from drying of the silicone gel phase (Figure 6b). 

**Figure 7:**
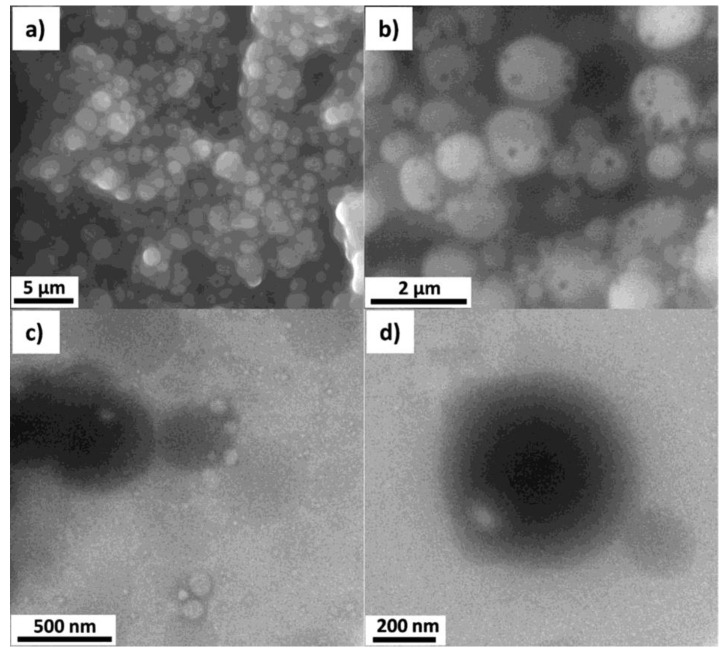
Morphology of the latex phase obtained with the siloxane oligomer and f = 0.8 conditions (sample EG7) observed by SEM (**a**,**b**) and TEM (**c**,**d**).

For compositions with f = 0.33 and f = 0.5, gradient emulsion structure [2] was frozen in the xerogels, with smaller pores on the surface and much larger pores on the bottom of the films or monoliths, which still preserve the multiple emulsion aspect (Figure 6c). In such a case, Janus membranes can be obtained in a one-step click reaction, since they present two surfaces with different morphology and properties. All the described materials were obtained based on a high molecular mass siloxane copolymer, which plays the role of a gelator, since it dissolves in toluene and ensures a relatively high viscosity, thus facilitating the stability of water droplets [5]. When this copolymer was replaced with a similar one with lower content of vinyl groups, and its proportion in the emulsion gel was diminished to half (sample EG10M), higher porosity was observed in the resulted monolith (Figure 6d). In another experiment, a long telechelic oligomer of M_n_~10,000 g/mol and vinyl end-groups was used instead of the copolymer. Thus, a less viscous mixture was obtained, and a looser cross-linking by chain ends. In order to increase the viscosity, a lower amount of toluene was used, and thus the proportion of water internal phase increased to f = 0.8. However, in this case (sample EG7), a phase separation occurred during UV cross-linking, leading to a thin film and a latex. The film had a wrinkled appearance in SEM, with some micron-size pores and small pores, preserving to a certain extent the morphology discussed above (Figure 6e,f). The latex evolved in a fragile material formed by cross-linked polymer particles, bearing nanopores (Figure 7a,b). The same complex morphology could be observed by TEM of a latex sample placed on a grid from the initial dispersion (Figure 7c,d). Water-in-oil droplets coexist with the latex particles and collapse on their surface by Ostwald ripening, creating small holes after water evaporation. 

**Figure 8:**
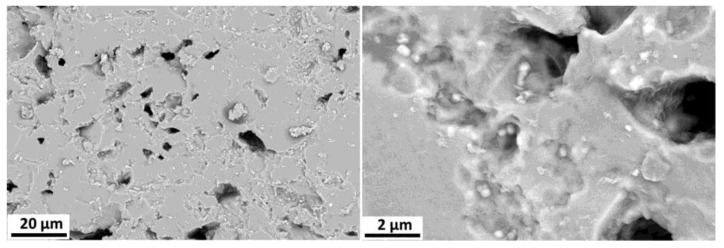
Polysiloxane–polyethyleneglycol porous composite obtained from an emulsion gel (sample EG4).

When the water phase contained PEG in 20% proportion reported to the siloxane matrix, submicron PEG particles were observed within the entire cross-section of the film, including in the pores (Figure 8). This is an easy and convenient way of obtaining all-polymer porous composites, from two incompatible polymers. The morphology of the composites (although not extensively studied yet) was different in the two examples shown in Figure 3e (prepared without co-surfactant) and in Figure 8. In the former case, the PEG particles were roughly around 1 micrometer in diameter, while in the latter these were around 200 nm. The difference comes from the particularities of each emulsion in the presence of polysiloxane. Thus, PEG was “filling” the large pores in the first example, and the small ones in the second, while the complementary set of pores remained open. Therefore, using or not using the co-surfactant is one way of controlling the composites’ morphology. In both cases, the PEG particles were relatively uniformly dispersed within the film thickness.

**Figure 9:**
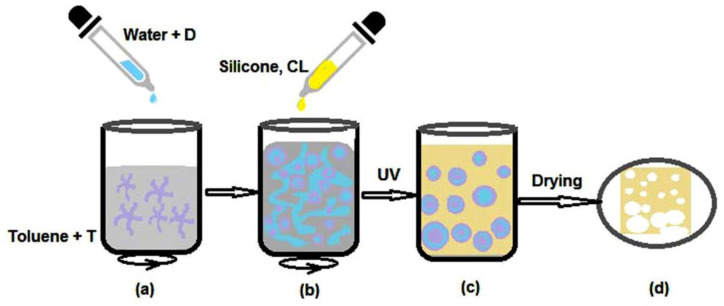
Schematic representation of emulsion templating preparation of porous silicone materials: (**a**) addition of aqueous solution of D into toluene solution of T surfactant; (**b**) formation of complex emulsion structures; (**c**) emulsion gel containing vinyl–polysiloxane, the thiol cross-linker and the initiator, cross-linked under UV irradiation; (**d**) porous monolith after drying. (The T surfactant and the w/o interface were drawn in purple, and the water phase in blue).

Taking into account the information from electron microscopy observations, we proposed a schematic representation of the entire process of pore formation (Figure 9). The initial system contains surfactant T dissolved in toluene (a). When water containing co-surfactant D is added, w/o emulsion starts to form spontaneously until an excess of aqueous phase accumulates and the micelles of D entrap toluene, forming o/w emulsion. According to TEM (Figure 4), several types of emulsion droplets coexist, so it is supposed that a bicontinuous phase is present, allowing formation of triple emulsions as well (b). At this point, the silicone mixture is added, i.e., the vinyl copolymer, the SH cross-linker and the photoinitiator (Scheme 2). Due to increased hydrophobicity of the silicone (which is basically a slightly modified PDMS, known for its very low surface tension), the water from the bicontinuous phase is forced into w/o′ droplets, and the proportion of o/w/o′ increases, wherein we denoted o′ as the “toluene + silicone” phase. These changes are suggested by the absence of interconnected large pores or cracks in the final materials (which would indicate a bicontinuous emulsion gel template) according to SEM. Next, the UV-initiated cross-linking occurs within a few minutes (c). During gelation in steady conditions, the larger droplets, containing more water, tend to accumulate at the bottom of the vessel, templating larger pores in the final materials with medium or high internal phase (d).

It is worth mentioning that the pore diameters were largely polydisperse in all the samples, as was estimated in Figure 6, with “large” pores of tens of microns and “smaller” pores, partly distributed inside the larger ones, due to particularities of these complex emulsions. We suppose that adjustments for more uniform pore size are possible by the level of the energy input, which would control the size of the droplets in the emulsion template. In the meantime, the polydispersity may be of interest for certain applications, as reported in the case of materials with gradient pore size for scaffolds and extracellular matrix [34,35]. Similar size and morphology of the pores and some adjustments in pore distribution by nature and concentration of the surfactants were reported by Abshirini et al. [36], for porous silicones obtained by hydrosilylation in emulsion templating. Even larger pores were reported for porous silicones (sponges or foams) obtained with solid NaCl [37] or supercritical CO_2_ [38] as templates. 

### 3.3. Brief Characterization of the Obtained Xerogels

Porous silicone materials are very interesting for a number of applications, including sensors, filtration membranes, delivery of active principles, or cell growth, to mention only a few. By using the concept developed in this work, very small amounts of silicone-containing surfactants are used in a simple method that allows various adjustments in order to tune the final properties according to the needs. For example, the cross-linking density can be adjusted by modifying the content of vinyl groups in the polysiloxane precursor, while the viscosity of the system depends (for a fixed phase ratio) on the molecular mass of the polysiloxane precursor. The amount of internal phase (water) influences the emulsion and monolith morphology, as well as the porosity of the materials. We expect that the rate of the photoinduced reaction, which can be adjusted by the structure and proportion of the thiol cross-linker and by the irradiation conditions, might also help optimize the high porosity products. The addition of complementary polymers (such as the hydrophilic PEG) opens a wide window for composite materials bearing porous structures at the same time. Another modification could be the replacement of toluene as the continuous phase with octamethylcyclotetrasiloxane (D4) or silicone oil, allowing the long-term preservation of the monolith in the swollen state, as tested here for sample EG9M.

Selected monoliths or films obtained herein were briefly characterized in order to verify the hypothesis of tunable properties. We have to mention that the materials were not optimized yet, since different properties are required for different applications, which will be developed in the future. 

Water contact angle (CA) measurements showed a dual character on some of the porous membranes, with very high contact angle on the upper side (where toluene rapid evaporation ensures an almost continuous silicone layer) and lower values on the bottom side (where in many cases larger pores form due to gravimetrical separation of water-reach droplets). Such a case is sample EG5, which can be regarded as a Janus membrane. Its surface behavior is in line with the observed morphology (Figure 6c). When a highly porous structure resulted, a pseudo-hydrophilic behavior was obtained on both sides, due to the rapid absorption of water in the pores (sample EG6). The values presented in Table 3 were read at the contact of the sessile drop with the surface. When observed in dynamic regime, the contact angle decreased very quickly, due to the porous structure, and air bubbles were visible once water penetrated the membrane, as can be seen in Figure S4 (gif file). If we compare a sample with PEG particles encapsulated with the corresponding one without PEG (EG4 vs. EG5), the difference in CA on both sides was not as pronounced in the former case as in the latter, due to a more compact texture, with pores “blocked” by PEG particles, according to SEM images. For a film that rapidly separated from the water-reach phase (EG8), the increased hydrophobic character of the polysiloxane manifested due to a lower level of porosity. 

Mechanical tests of the films were conducted in stress–strain setup and the Young’s modulus was calculated in each case, showing values lower than 1 MPa, with a general decreasing trend with an increase in porosity (Table 4), similar as reported by other authors [10]. Low maximum strain was a characteristic of these porous materials, since large pores act as mechanical defects in this kind of test. Nevertheless, flexible films were obtained, and the mechanical properties are largely tunable by proper choice of the starting vinyl–silicone. In fact, when the same silicone precursor was cross-linked in the same conditions, without the emulsion gel transient state, its mechanical properties were worse; the film was brittle and could not withstand typical stress–strain measurements. When a silicone precursor with low content of vinyl groups was used (EG8), its Y modulus was lower than that of the similar sample with high cross-linking density (EG6), but a more pronounced separation of the film after UV irradiation was observed for the same composition. On the other hand, the monolith obtained with Co-1.8 (EG8M) had poor mechanical resistance, so it was not further tested. 

**Figure 10:**
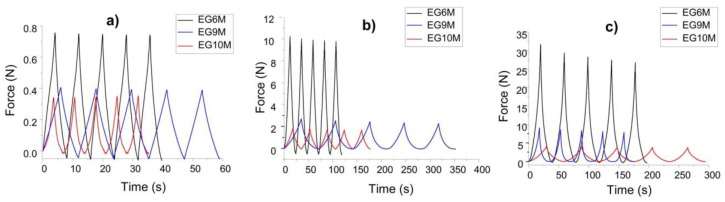
Compression tests of the monoliths at different compressive strains: (**a**) 10%, (**b**) 30%, and (**c**) 50%.

The other three monoliths, prepared from f = 0.5 compositions with both siloxane copolymers in different proportion, were tested in compression mode, either in dry or swollen state (Video S1—video). Satisfactory results were obtained in this stage, for non-optimized soft materials. The samples withstood important compression force and resisted several cycles without damage (Figure 10). The Young’s modulus of the monoliths, measured in compression mode, was one order of magnitude lower than those of the films, measured by tensile tests. When the two monoliths analyzed in swollen state are compared (EG9M and EG10M), lower Y was obtained for the more porous one, but the nature of the swelling liquid is also important. It was found that the monolith swollen in D4 required less effort for the same compression degree, which would mean that D4 has a lubricating effect, due to its high affinity with the matrix. Such materials will be optimized for pressure sensors.

**Figure 11:**
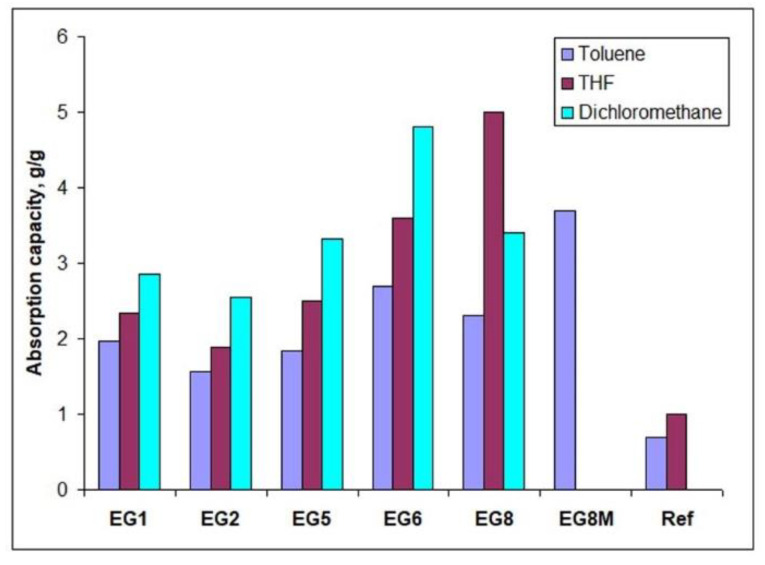
Absorption capacity (g/g) of xerogels after 30 min of immersion in different solvents.

Swelling experiments were done in three different solvents and the results are presented in Figure 11. The absorption capacity was relatively high, compared with the silicone reference (without pores) and generally increased with the internal phase fraction (porosity). Values close to 500% could be obtained in certain cases. Higher absorption capacity for toluene was measured for a monolith (EG8M) compared with the corresponding film (EG8). On the other hand, this monolith did not preserve its integrity after the absorption experiment. 

## 4. Conclusions

Two compounds containing short (D) or long (T) dimethylsiloxane segments, and terminal pyridyl groups, designed to be soluble in water or toluene, respectively, were investigated for interfacial properties and emulsions stabilization. Negative interfacial tension values were measured by Wilhelmy plate tensiometry, and quasi-spontaneous emulsification was observed with 0.1% and 1% toluene solution of T. By adding vinyl-containing polysiloxanes to the formed emulsion, followed by photo-initiated cross-linking, emulsion gels were obtained, which were the templates for silicone xerogels. When only T was used as a stabilizer, a low water internal phase could be introduced. By using an aqueous solution of D as a co-surfactant, a stable emulsion with complex structure and a medium or high internal phase was formed by gentle manual mixing and brief sonication in a cleaning bath. The resulted porous materials preserved the morphology of the emulsion template. The presence of a second polymer (PEG), dissolved in the aqueous phase, allowed in situ formation of submicron particles embedded into the silicone matrix, thus opening a novel perspective for easy, one-pot preparation of all-polymer composites from incompatible polymer pairs. 

Materials with tunable (surface, mechanical, absorption) properties can be obtained by this method, and promising applications are envisaged, e.g., Janus membranes or pressure sensors. 

## Figures and Tables

**Table 1:** Components of the emulsion gels.

Code	Surfactants, Conc.	Silicone Type, Conc. (*w*/*v*) ^a^	Water Content (f) ^b^	Additive, Conc. ^c^/ Other Conditions
PE1	T, 1 g/L	Co-8.5, 20%	0.1	
PE2	T, 1%	Co-8.5, 20%	0.16	
PE3	T, 1%	Co-8.5, 20%	0.1	PEG, 10%
EG1	T, 1% and D, 1%	Co-8.5, 20%	0.16	
EG2	T, 1% and D, 1%	Co-8.5, 20%	0.25	
EG3	T, 1% and D, 1%	Co-8.5, 20%	0.25	PEG 10%
EG4	T, 1% and D, 1%	Co-8.5, 20%	0.33	PEG 20%
EG5	T, 1% and D, 1%	Co-8.5, 20%	0.33	
EG6	T, 1% and D, 1%	Co-8.5, 20%	0.5	Very porous film separated
EG6M				Monolith
EG7	T, 5% and D,1%	O, 110%	0.82	Phase separation: thin film and latex
EG8	T, 1% and D, 1%	Co-1.8, 20%	0.5	Film separated from liquid
EG8M				Monolith
EG9M	T, 1% in D4 and D, 1%	Co-1.8, 20%	0.5 (vs. D4)	Monolith
EG10M	T, 1% and D, 1%	Co-1.8, 10%	0.5	Monolith

^a^ Reported to toluene; ^b^ volume fraction, f = V_water_/V_(toluene + water)_; ^c^ reported to silicone.

**Table 2:** Surface properties of 1 g/L solutions of the two amphiphilic siloxane compounds used as stabilizers.

Sample	Surface Tension, γ (mN/m)	Interfacial Tension, σ (mN/m)	Surface Tension of Emulsion, γ (mN/m)
D (water)	35.65 (±0.27)	−4.21 (±0.3)	26.37 (±3.5) o/w
T (toluene)	25.28 (±0.025)	−2.95 (±0.15)	25.33 (±0.21) w/o 25.91 (±0.9) o/w

**Table 3:** Water contact angles measured on both sides of selected porous films.

Sample	CA Upper Side (°)	CA Bottom Side (°)
EG4	117.2 ± 6.5	93.8 ± 3.1
EG5	111.7 ± 4.6	66.8 ± 5.8
EG6	75 ± 18	73.7 ± 14
EG8	124.3 ± 6.1	97.1 ± 6.3

**Table 4:** Young’s modulus of the porous silicone films measured on stress–strain and for monoliths in compression mode.

Code	Composition	Y, MPa	Obs.
EG1	f = 0.16	0.64	film
EG2	f = 0.25	0.70	film
EG3	f = 0.25; PEG 10%	0.65	film
EG4	f = 0.33; PEG 20%	0.49	film
EG5	f = 0.33	0.44	film
EG6	f = 0.5	0.45	film
EG8	f = 0.5 (Co-1.8)	0.33	film
EG6M	f = 0.5 (Co-8.5)	0.032	Dried monolith
EG9M	f = 0.5 (Co-1.8; D4)	0.010	Monolith swollen in D4
EG10M	f = 0.5 (Co-1.8, 10%)	0.008	Swollen monolith

## Data Availability

The data presented in this study are available in the article and Supplementary Files.

## Supplementary Materials

The following supporting information can be downloaded at: https://www.mdpi.com/article/10.3390/gels8060377/s1. Figure S1: Interfacial tension of aqueous solutions of D versus toluene, measured with Wilhelmy plate, in “surface/interfacial tension” (manual) and “continuous Wilhelmy plate” measurement modes; Figure S2: Emulsification of 1 g/L toluene solution of T with 37.5% water: (a) initial aspect of w/o and o/w emulsions after sonication with an ultrasonic processor (VC 505); (b) the aspect of the same sample after 24 h of standing (o/w emulsion stabilized); (c) result of manual stirring of (b) after one week of standing; Figure S3: Silicone gels obtained from the w/o emulsion, using as continuous phase a polysiloxane toluene solution containing 1 g/L surfactant T and ca. 10% water as internal phase; Figure S4—dynamic CA (gif image); Video S1—compressive test (video).
