# Supplementary material for: Emulsion Gels as Precursors for Porous Silicones and All-Polymer Composites—A Proof of Concept Based on Siloxane Stabilizers
Source: Gels. 2022 Jun 14;8(6):377. doi: 10.3390/gels8060377 (PMC9222695; doi:10.3390/gels8060377)
Supplement: Supplementary file 1 [file gels-08-00377-s001.zip › gels-1742165- supplementary.pdf]

gels

MDPI

Supplementary Matgerials

Emulsion Gels as Precursors for Porous Silicones and All-Polymer Composites - A Proof of Concept Based on Siloxane Stabilizers

Carmen Racles [1,*], Adrian Bele [1], Ana-Lavinia Vasiliu [2] and Liviu Sacarescu [1]

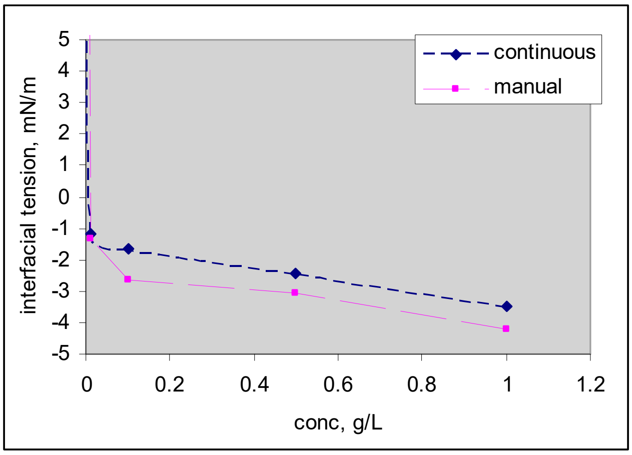

Figure S1. Interfacial tension of aqueous solutions of D versus toluene, measured with Wilhelmy plate, in “surface/interfacial tension” (manual) and “continuous Wilhelmy plate” measurement modes.

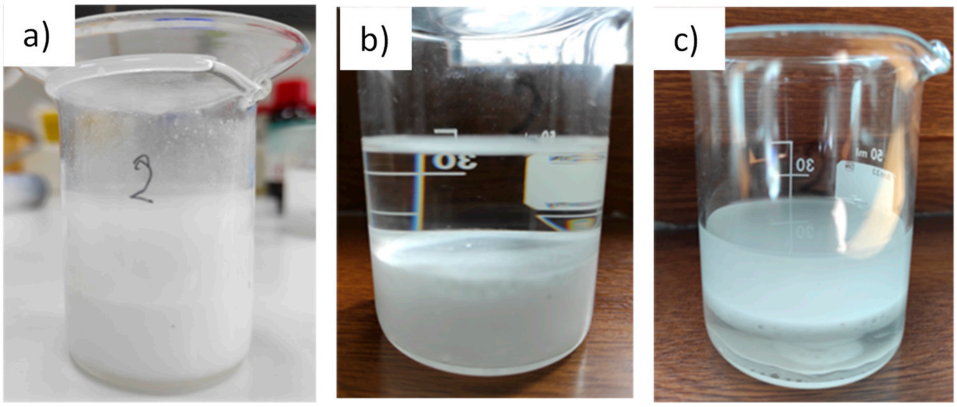

Figure S2. Emulsification of 1g/L toluene solution of T with 37.5% water: a) initial aspect of w/o and o/w emulsions after sonication with an Ultrasonic processor (VC 505); b) the aspect of the same sample after 24h of standing (o/w emulsion stabilized); c) result of manual stirring of (b) after one week of standing.

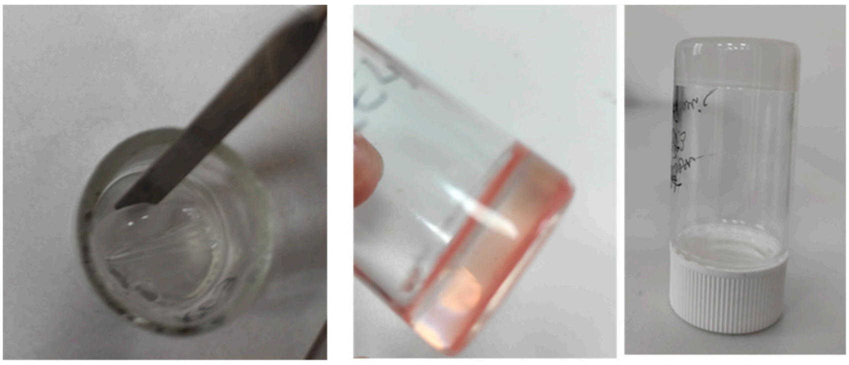

Figure S3. Silicone gels obtained from the w/o emulsion, using as continuous phase a polysiloxane toluene solution containing 1g/L surfactant T, and ca. 10% water as internal phase.
