# Supplementary figures and images for: Emulsion Gels as Precursors for Porous Silicones and All-Polymer Composites—A Proof of Concept Based on Siloxane Stabilizers
Source: Gels. 2022 Jun 14;8(6):377. doi: 10.3390/gels8060377 (PMC9222695; doi:10.3390/gels8060377)

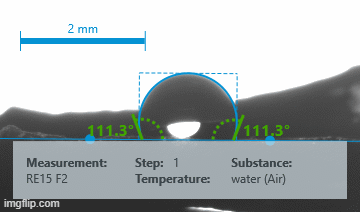

Supplement: Supplementary file 1 [file gels-08-00377-s001.zip › gels-1742165- supplementary-Figure S4.gif]
